# Outcomes of patients hospitalized for acute decompensated heart failure: does nesiritide make a difference?

**DOI:** 10.1186/1471-2261-7-37

**Published:** 2007-11-26

**Authors:** Richard J Carroll, Zuber D Mulla, Loran D Hauck, Audrey Westbrook

**Affiliations:** 1 Adventist Health System, LaGrange, Illinois, USA; 2 Texas Tech University Health Sciences Center Paul L. Foster School of Medicine, El Paso, Texas, USA, and University of Texas School of Public Health at Houston, El Paso, Texas, USA; 3 Adventist Health System, Orlando, Florida, USA; 4 Florida Hospital Memorial System, Ormond Beach, Florida, USA

## Abstract

**Background:**

Nesiritide is indicated in the treatment of acute decompensated heart failure. However, a recent meta-analysis reported that nesiritide may be associated with an increased risk of death. Our goal was to evaluate the impact of nesiritide treatment on four outcomes among adults hospitalized for congestive heart failure (CHF) during a three-year period.

**Methods:**

CHF patients discharged between 1/1/2002 and 12/31/2004 from the Adventist Health System, a national, not-for-profit hospital system, were identified. 25,330 records were included in this retrospective study. Nesiritide odds ratios (OR) were adjusted for various factors including nine medications and/or an APR-DRG severity score.

**Results:**

Initially, treatment with nesiritide was found to be associated with a 59% higher odds of hospital mortality (Unadjusted OR = 1.59, 95% confidence interval [CI]: 1.31–1.93). Adjusting for race, low economic status, APR-DRG severity of illness score, and the receipt of nine medications yielded a nonsignificant nesiritide OR of 1.07 for hospital death (95% CI: 0.85–1.35). Nesiritide was positively associated with the odds of prolonged length of stay (all adjusted ORs = 1.66) and elevated pharmacy cost (all adjusted ORs > 5).

**Conclusion:**

In this observational study, nesiritide therapy was associated with increased length of stay and pharmacy cost, but not hospital mortality. Randomized trials are urgently needed to better define the efficacy, if any, of nesiritide in the treatment of decompensated heart failure.

## Background

Congestive heart failure (CHF) is a significant public health concern and pressing public policy issue. With greater than 5 million patients who carry the diagnosis of CHF in the United States alone, and with approximately 550,000 new cases per year, it is no surprise that acute decompensated heart failure (ADHF) is the leading cause of hospitalization in the U.S. in persons over the age of 65 years [1,2]. Hospital discharges for CHF rose from 377,000 in 1979 to 970,000 in 2002, an increase of 157%. In 2005 it is estimated that $27.9 billion will be spent on direct costs for CHF, $14.7 billion on hospital care, and $2.9 billion on drugs/other medical durables [1]. With hospital readmission rates as high as 50% at six months, and with the major medical society guidelines focused on the outpatient management of CHF, physicians are always looking for innovative approaches as well as guidance on how to best manage patients with ADHF [3-5].

In 2001 nesiritide (Natrecor^®^) was approved by the FDA for the intravenous treatment of patients with ADHF who have dyspnea at rest or with minimal activity, based on data which showed nesiritide reduced pulmonary capillary wedge pressure and improved dyspnea [6]. Given the plausible biological mechanism of action of nesiritide and its safety profile as compared to existing intravenous inotropic agents, nesiritide use rapidly increased [7-9]. However, two recent publications have questioned the safety of nesiritide in terms of increased mortality and worsening renal function in patients treated with this drug for acutely decompensated heart failure [10,11]. At one point physicians at the Cleveland Clinic, one of the nation's largest cardiac centers, considered severely curtailing or even banning the clinic's use of nesiritide [12]. Subsequently, a panel of cardiologists and heart failure experts was convened, chaired by Dr. Eugene Braunwald, to review the data associated with nesiritide and made recommendations on its use. Reviewing the published data along with other information available on nesiritide, the panel recommended strictly limiting the use of nesiritide to patients presenting to the hospital with ADHF who have dyspnea at rest, and recommend continued enrollment in ongoing trials of nesiritide, as well as pro-active educational programs to inform physicians regarding conditions and circumstances in which nesiritide should and should not be used [13].

Adventist Health System (AHS) is a multi-hospital, not-for-profit, health care system with community hospitals in many geographic locations. It has over 8,000 admissions per year for CHF. Given the volume of patients seen for CHF and the exponential growth in the use of nesiritide at our facilities, we decided to review our data over a three year period (2002 to 2004; representing more than 25,000 patient encounters) looking specifically at both the clinical outcomes and financial impact of nesiritide use.

## Methods

### Patient Population

Data from 31 of the AHS hospitals were electronically transferred to a clinical decision support software vendor MEDai (Medical Artificial Intelligence, Inc., Orlando, Florida). Discharge diagnoses were coded using the *International Classification of Diseases, Ninth Revision, Clinical Modification *(ICD-9-CM).

Our study included electronic records of patients who were 18 years of age or older who were discharged for CHF between January 1, 2002, and December 31, 2004. CHF was defined as the occurrence of one of the following ICD-9-CM codes in the primary diagnosis field: 402.01, 402.11, 402.91, 404.01, 404.03, 404.11, 404.13, 404.91, 404.93, 428.0, 428.1, 428.9.

Records were excluded if they had missing values for the dependent or independent variables under study. A total of 25,330 records were included in this study. These records do not represent 25,330 unique patients since some patients were readmitted during the study period for CHF. This sample included 18,298 distinct patients.

### Primary Statistical Analysis

The exposure of interest was receipt of nesiritide during the hospital stay: Patients who received nesiritide were compared to patients who did not receive nesiritide. Four outcomes were studied: hospital mortality, prolonged length of stay (defined below), elevated pharmacy cost (defined below), and readmission to an AHS hospital within 31 days for a Diagnosis Related Group (DRG) in Major Diagnostic Category 5, diseases and disorders of the circulatory system (as defined by the U.S. Centers for Medicare and Medicaid Services).

Prolonged length of stay was defined as a length of stay greater than the 75^th ^percentile, > 6 days in our study. Elevated pharmacy cost was defined as a pharmacy cost greater than the 75^th ^percentile, > $938 in our study. Previous studies in the area of clinical epidemiology have used the 75^th ^percentile to dichotomize a continuous variable [14,15].

The SAS System Release 8.02 (SAS Institute, Inc., Cary, North Carolina) was used to perform logistic regression. Crude and adjusted odds ratios (OR) for the four outcomes were calculated along with 95% confidence intervals (CI) using PROC GENMOD. Two methods were used to control for confounding by disease severity: Multivariate modeling, and stratification with multivariate modeling. Initially, the nesiritide odds ratio was adjusted for the following two disease severity measures (one at a time) using a logistic regression model: All Patient Refined-Diagnosis Related Group (APR-DRG) severity of illness score, and the APR-DRG risk of mortality score [16]. These two patient classification systems incorporate a variety of demographic and clinical information including age, sex, diagnoses, and procedures. The final models included one of the two severity scores, a race/ethnicity variable (White, Black, Hispanic, and Other), a variable identifying low economic status (yes/no), and the following nine medications: angiotensin converting enzyme inhibitors, angiotensin II receptor antagonists, digoxin, diuretics, dobutamine, dopamine, beta-blockers, intravenous nitroglycerin, and milrinone. Multicollinearity was not detected in these final/full models.

In the second approach, odds ratios for hospital mortality were stratified by the two different severity measures: APR-DRG severity of illness score, and the APR-DRG risk of mortality score. Analyses of the remaining three outcomes were stratified by the APR-DRG severity of illness score. In each of the strata the OR was adjusted for the receipt of the following medications: angiotensin converting enzyme inhibitors, angiotensin II receptor antagonists, digoxin, diuretics, dobutamine, dopamine, beta-blockers, intravenous nitroglycerin, and milrinone.

Both of the APR-DRG measures are ordinal variables ranging from 1 to 4. Due to the small number of deaths and episodes of prolonged length of stay among patients classified as APR-DRG severity level 1 or APR-DRG risk of mortality level 1, several of the logistic regression models in the stratified analysis failed to converge. To counteract this problem we collapsed levels 1 and 2 into one category for both APR-DRG indices. We did not alter the APR-DRG indices for our initial multivariate analyses.

### Repeated Measurements

The inclusion of multiple records for certain patients required a method to account for the correlated nature of the data. Generalized estimating equations (GEE) were used to address this issue [17]. GEE was used to calculate robust standard errors leading to appropriate 95% confidence intervals for the population odds ratios. An exchangeable correlation structure was specified [18].

### Secondary Statistical Analysis

To ensure a more homogenous study sample only unique patients were included in a secondary analysis. To clarify, the primary analysis included a group of patients who were admitted multiple times for CHF during the study period. It is likely that these individuals were sicker than CHF patients who were only admitted once during the three-years of observation and therefore more likely to receive nesiritide. To minimize the risk of confounding by disease severity a secondary analysis was performed in which only the record of the initial episode of care was retained if the patient had two or more admissions for CHF. There were 18,195 patients who fell into this category after deleting records with missing values.

Crude and adjusted ORs associated with inpatient nesiritide treatment were calculated using PROC LOGISTIC in SAS. The following four outcomes were studied: hospital mortality, prolonged length of stay (defined below), elevated pharmacy cost (defined below), and readmission to an AHS hospital within 31 days for a cardiac/circulatory system disorder other than CHF. Prolonged length of stay was defined as a length of stay greater than the 75^th ^percentile, > 6 days in our study. Elevated pharmacy cost was defined as a pharmacy cost greater than the 75^th ^percentile, > $928 in our study.

The study protocol was approved by the Committee for the Protection of Human Subjects at The University of Texas Health Science Center at Houston.

## Results

**Figure 1:**
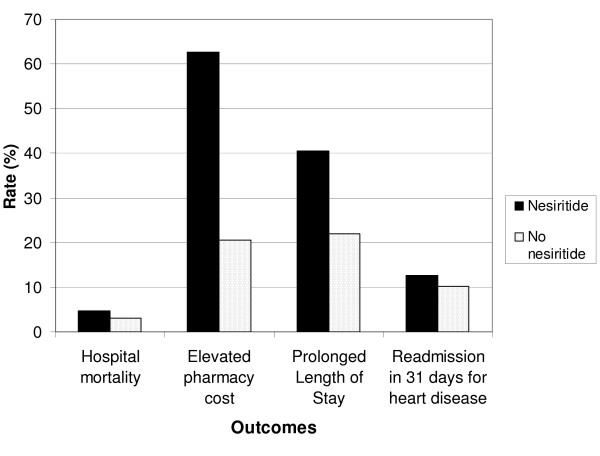
Unadjusted rates of four outcomes by nesiritide status.

Approximately 11% of the CHF cohort (2692 patients) received nesiritide during their hospital stay (Table 1). Table 1 indicates that the patients who received nesiritide were more severely ill than the patients who did not receive nesiritide. Overall, 805 of the 25,330 patients expired in the hospital (Table 1). The distribution of the deaths was as follows: 126 patients who received nesiritide, and 679 patients who did not receive nesiritide (Table 1). The crude hospital mortality rate among patients who received nesiritide was 57% higher than the hospital mortality rate among patients who did not receive nesiritide: 4.7% versus 3.0% (Relative risk = 1.57) (Figure 1, and Table 1).

**Table 1:** Demographic and Clinical Characteristics of 25,330 Patients Hospitalized for CHF

Variable	Received nesiritide (n = 2692)	Did not receive nesiritide (n = 22,638)
	Number (%)	Number (%)
Demographic variables*		
Race/Ethnicity		
Black	292 (10.9)	2438 (10.8)
Hispanic	266 (9.9)	1570 (6.9)
White	2125 (78.9)	18,512 (81.8)
Other race	9 (0.3)	118 (0.5)
Low economic status	118 (4.4)	960 (4.2)
		
**Medications**		
ACE inhibitors	1558 (57.9)	12,173 (53.8)
Angiotensin II receptor antagonists	574 (21.3)	3478 (15.4)
Diuretics	2658 (98.7)	21,650 (95.6)
Digoxin	1320 (49.0)	8925 (39.4)
Beta blockers	2114 (78.5)	13,849 (61.2)
Dobutamine	341 (12.7)	1131 (5.0)
Dopamine	315 (11.7)	1185 (5.2)
IV nitroglycerin	313 (11.6)	1762 (7.8)
Milrinone	150 (5.6)	272 (1.2)
		
**Severity measures**		
APR-DRG Severity of Illness		
1 or 2	1343 (49.9)	14,653 (64.7)
3	1077 (40.0)	6979 (30.8)
4	272 (10.1)	1006 (4.4)
APR-DRG Risk of Mortality		
1 or 2	1618 (60.1)	16,716 (73.8)
3	781 (29.0)	4661 (20.6)
4	293 (10.9)	1261 (5.6)
		
**Outcomes**		
Hospital mortality	126 (4.7)	679 (3.0)
Prolonged length of stay	1088 (40.4)	4973 (22.0)
Elevated pharmacy cost	1688 (62.7)	4640 (20.5)
Readmission within 31 days for cardiac condition	340 (12.6)	2318 (10.2)

* Median age of patients who received nesiritide (range) = 74 years (20–100 years), median age of patients who did not receive nesiritide (range) = 76 years (18–105 years)

Nesiritide treatment was associated with a 59% higher odds of hospital mortality (crude OR = 1.59, 95% CI: 1.31–1.93) (Table 2). The crude relative risk for hospital death (1.57) and the crude odds ratio for hospital death (1.59) are similar indicating that the outcome of hospital mortality was not a common event. Adjusting for either of the APR-DRG measures attenuated the nesiritide ORs for hospital mortality and resulted in ORs that were not statistically significant. Crude and adjusted ORs revealed that treatment with nesiritide was associated with higher odds of prolonged length of stay and elevated pharmacy cost.

**Table 2:** Crude and Adjusted Odds Ratios for Four Outcomes: Nesiritide compared to No Nesiritide (n = 25,330)

	Outcome			
Type of odds ratio	Hospital mortality OR^† ^(95% CI^‡^)	Prolonged length of stay OR (95% CI)	Elevated pharmacy cost OR (95% CI)	Readmission within 31 days for cardiac condition OR (95% CI)

Unadjusted	1.59 (1.31–1.93)	2.41 (2.21–2.62)	6.24 (5.72–6.80)	1.11 (0.89–1.39)
Adjusted for 9 medications*	1.26 (1.00–1.58)	1.89 (1.72–2.06)	5.35 (4.88–5.86)	1.14 (0.90–1.44)
Adjusted for APR-DRG severity of illness score	1.04 (0.85–1.27)	1.99 (1.82–2.19)	6.24 (5.67–6.87)	1.14 (0.91–1.42)
Adjusted for 9 medications, APR-DRG severity of illness score, race, and low economic status	1.07 (0.85–1.35)	1.66 (1.51–1.83)	5.45 (4.93–6.01)	1.15 (0.91–1.45)
Adjusted for APR-DRG risk of mortality score	1.03 (0.84–1.27)	N/A	N/A	N/A
Adjusted for APR-DRG risk of mortality score, 9 medications, race, and low economic status	1.06 (0.84–1.35)	N/A	N/A	N/A

* Listed in Table 1

† OR = odds ratio

‡ CI = confidence interval

Stratifying by APR-DRG severity of illness did not reveal any associations between nesiritide and hospital mortality and nesiritide and readmission within 31 days for a cardiac condition (Table 3). Increased odds of prolonged length of stay and elevated pharmacy cost were linked to nesiritide treatment (Table 3).

**Table 3:** Odds Ratios* for Four Outcomes Stratified by APR-DRG Severity of Illness: Nesiritide compared to No Nesiritide

APR-DRG severity of illness score	Hospital mortality OR^† ^(95% CI^‡^)	Prolonged length of stay OR (95% CI)	Elevated pharmacy cost OR (95% CI)	Readmission within 31 days for cardiac condition OR (95% CI)
1 or 2 (n = 15,996)	1.22 (0.70–2.12)	1.66 (1.43–1.93)	6.45 (5.67–7.33)	1.09 (0.79–1.50)
3 (n = 8056)	0.89 (0.62–1.28)	1.70 (1.49–1.95)	4.63 (3.98–5.39)	1.19 (0.94–1.51)
4 (n = 1278)	1.12 (0.79–1.60)	1.62 (1.15–2.28)	4.44 (2.84–6.95)	(model failed)

Total (n = 25,330)				

* Adjusted for the nine medications listed in Table 1

† OR = odds ratio

‡ CI = confidence interval

A large number of patients were classified as having an APR-DRG risk of mortality of 1 or 2 (Table 4). After stratifying by the APR-DRG risk of mortality score, nesiritide neither significantly increased nor decreased the odds of mortality.

**Table 4:** Odds Ratios* for Hospital Mortality Stratifiedby APR-DRG Risk of Mortality: Nesiritide compared to No Nesiritide

APR-DRG risk of mortality score	Odds ratio (95% confidence interval)
1 or 2 (n = 18,334)	1.20 (0.69–2.10)
3 (n = 5442)	0.87 (0.59–1.27)
4 (n = 1554)	1.18 (0.85–1.64)

Total (n = 25,330)	

* Adjusted for the nine medications listed in Table 1

In a secondary analysis the sample was restricted to only one record per patient. If the patient was admitted more than once for CHF during the three-year study period then only the initial episode of care was retained. Unadjusted and adjusted ORs for the four outcomes of interest are shown in Table 5 for these 18,195 unique patients. The unadjusted hospital mortality OR (Table 5) was slightly attenuated compared to its counterpart in Table 2 but was still statistically significant. The four ORs for readmission within 31 days for a cardiac condition/disorder of the circulatory system other than CHF (Table 5) were higher than the readmission ORs in Table 2 and were significant at the 0.05-level unlike the readmission ORs in Table 2.

**Table 5:** Crude and Adjusted Odds Ratios for Four Outcomes in a Sample of CHF Patients with Only One Record per Patient*: Nesiritide compared to No Nesiritide (n = 18,195 unique patients)

	Outcome			
Type of odds ratio	Hospital mortality OR^† ^(95% CI^‡^)	Prolonged length of stay OR (95% CI)	Elevated pharmacy cost OR (95% CI)	Readmission within 31 days for cardiac condition other than CHF OR (95% CI)

Unadjusted	1.48 (1.14–1.93)	2.51 (2.26–2.79)	6.82 (6.12–7.61)	1.25 (1.05–1.49)
Adjusted for 9 medications**	1.23 (0.91–1.65)	1.97 (1.76–2.20)	5.78 (5.15–6.48)	1.25 (1.05–1.49)
Adjusted for APR-DRG severity of illness score	0.87 (0.66–1.15)	1.97 (1.76–2.22)	6.60 (5.85–7.43)	1.26 (1.06–1.50)
Adjusted for 9 medications, APR-DRG severity of illness score, race, and low economic status	0.98 (0.72–1.34)	1.65 (1.46–1.86)	5.66 (5.01–6.40)	1.25 (1.04–1.49)
Adjusted for APR-DRG risk of mortality score	0.89 (0.68–1.18)	N/A	N/A	N/A
Adjusted for APR-DRG risk of mortality score, 9 medications, race, and low economic status	1.01 (0.74–1.37)	N/A	N/A	N/A

* This sample excluded the subsequent records of patients who were admitted multiple times for CHF during the study period

** Listed in Table 1

† OR = odds ratio

‡ CI = confidence interval

## Discussion

Several recent events caused our health system to look at our own internal data regarding outcomes when nesiritide is used to treat patients with ADHF: 1. Several recent studies suggesting a negative impact of nesiritide on mortality and renal function [10,11], 2. A major academic medical center expressing concerns regarding nesiritide use [12], 3. A federal subpoena issued regarding marketing strategies for this medication [19], 4. The need for a national panel of cardiology and heart failure experts to re-review data regarding outcomes of patient's treated with nesiritide [13], and 5. Our own corporate data suggesting significant increases in the use of nesiritide.

Our post-marketing surveillance study found that after accounting for various clinical and demographic variables, there was no relationship between nesiritide and hospital mortality. Our preliminary analysis used a slightly different set of potential confounders and found that treatment with nesiritide was associated with a 26% increase in the odds of hospital mortality (Adjusted incidence odds ratio = 1.26) [20,21]. Even though this result was statistically significant (p = 0.03) it was barely so as evidenced by a 95% CI that almost contained the null value of one: 1.02–1.57. In a meta-analysis of clinical trial data, Sackner-Bernstein *et al*. [10] found a risk ratio of death within 30 days of 1.74 for patients randomized to nesiritide (95% CI: 0.97–3.12) and a hazard ratio after adjusting for study of 1.80 (95% CI: 0.98–3.31). A revised study by Aaronson and Sackner-Bernstein revealed adjusted relative risks of approximately 1.9 for mortality within 30 days of treatment for nesiritide versus control therapy [22]. These relative risks were statistically significant, but, nonetheless, the authors caution that their studies were not designed to conclusively determine if nesiritide is associated with the risk of death [22].

Nesiritide was associated with a prolonged length of stay in the hospital and increased pharmacy cost in our investigation. The cost data are disturbing, although not completely surprising. Since January 2003 nesiritide has ranked among the top 10 medications contributing to overall AHS corporate medication costs. For 2005 year to date, it ranks as the third most expensive medication across our corporation despite being used in only 11% of our CHF admissions. However, the magnitude of the elevated pharmacy costs (adjusted odds ratios of greater than 5 for the nesiritide treated group) was unexpected. The obvious question at this point is whether or not this significantly increased cost is warranted, particularly given the increased length of stay.

We believe this retrospective review of data from geographically dispersed hospitals in our health system is probably representative of how nesiritide is used across the USA in community hospitals as opposed to academic medical centers where research trials are typically conducted. Even though our data are not from a clinical trial or an industry-sponsored registry our dataset was similar to the ADHERE Registry with respect to the use of nesiritide: 11% of our patients were treated with nesiritide while 12% of the patients in the ADHERE Registry were on nesiritide [23]. Our use of a non-industry-sponsored dataset can be viewed as an advantage since industry-sponsored patient registries may suffer from sampling bias. The three-year period for this observational study allowed us to include over 25,000 patient encounters including over 2,600 patients that received nesiritide. In comparison, the VMAC trial enrolled 489 patients, 204 of whom received nesiritide [24].

A retrospective study has several limitations. Our data set did not allow us to determine when or where nesiritide therapy began and for how long the infusion was continued. We were also unable to obtain salient clinical parameters such as etiology of the heart failure, left ventricular ejection fraction on admission, serum brain natriuretic peptide levels, pulmonary capillary wedge pressure readings, or other estimates of the degree of volume overload. Such variables may have increased the sensitivity of our severity adjustments. Some authors have suggested reserving nesiritide for only the most seriously ill patients. However, our data, when stratified by severity level (Tables 3 and 4), would suggest that the sickest patients did not benefit more with nesiritide as anticipated. Also, our dataset was unable to determine whether or not the addition of nesiritide improved diuresis.

Our study was not a randomized controlled trial; however, it would have been challenging to enroll as many patients as we had (over 18,000) in a clinical trial. Furthermore, randomized clinical trials offer insights into the efficacy of an intervention but not necessarily the effectiveness of an intervention. In other words, clinical trials are conducted under somewhat artificial conditions while observational studies take place in the "real" world.

The principal limitation of this study is the observational design and the fact that it is known that sicker patients receive nesiritide. The study design cannot overcome unmeasured confounding or a bias from the inclusion of a select group of severe patients who may have been channeled to the therapy under investigation. Therefore the results are ultimately hypothesis generating. However, the results of this study provide clear impetus for randomized controlled trials to define the role of nesiritide in the treatment of heart failure.

If nesiritide is a useful agent in the treatment of ADHF, the conditions under which it has proven utility must be documented and defined. While lowering pulmonary capillary wedge pressure and improving dyspnea are advantageous in ADHF, they alone do not warrant the increased cost of nesiritide use. This is particularly true when other outcome measures such as length of stay in our study and survival and renal function in other studies [10,11] have shown disturbing trends.

We add our voice to those others who seek data from randomized, controlled clinical trials as to the utility for nesiritide in the treatment of ADHF [13,25].

## Conclusion

In this observational study, nesiritide therapy was associated with increased length of stay and pharmacy cost, but not hospital mortality. Randomized trials are urgently needed to better define the efficacy, if any, of nesiritide in the treatment of decompensated heart failure.

## List of Abbreviations

ADHF = acute decompensated heart failure

AHS = Adventist Health System

APR-DRG = All Patient Refined-Diagnosis Related Group

CHF = congestive heart failure

CI = confidence interval

OR = odds ratio

## Competing interests

The author(s) declare that they have no competing interests.

## Authors' contributions

RJC, LDH, and AW conceived the initial research questions. All authors participated in the design of the study. ZDM analyzed the data and received compensation from Adventist Health System for his time. All authors participated in the interpretation of the results and the drafting and approval of the manuscript.

## Pre-publication history

The pre-publication history for this paper can be accessed here:


